# Supplementary material for: ProteinShader: illustrative rendering of macromolecules
Source: BMC Struct Biol. 2009 Mar 30;9:19. doi: 10.1186/1472-6807-9-19 (PMC2672931; doi:10.1186/1472-6807-9-19)
Supplement: Additional file 1 — ProteinShader program without source code. This compressed file contains the complete ProteinShader program including associated libraries, but no source code. A README.txt file gives an overview of the ProteinShader distribution, and the index.html file in the help subdirectory has directions on getting started with the program as well as a set of tutorials. [file 1472-6807-9-19-S1.zip › ProteinShader-beta-0_9_4-binary/help/api/org/proteinshader/structure/enums/BondEnum.html]

BondEnum (ProteinShader API)


|  |  |  |
| --- | --- | --- |
| **Overview** \| **Package** \| **Class** \| **Use** \| **Tree** \| **Deprecated** \| **Index** \| **Help** | |  |
| **PREV CLASS**   **NEXT CLASS** | **FRAMES**    **NO FRAMES**     **All Classes** |  |
| SUMMARY: NESTED \| ENUM CONSTANTS \| FIELD \| METHOD | DETAIL: ENUM CONSTANTS \| FIELD \| METHOD |  |


---


## org.proteinshader.structure.enums Enum BondEnum

```
java.lang.Object
  java.lang.Enum<BondEnum>
      org.proteinshader.structure.enums.BondEnum
```

**All Implemented Interfaces:**: Serializable, Comparable<BondEnum>

---

``` public enum BondEnum extends Enum<BondEnum> ```

Provides an enumeration for molecular bonds. Each BondEnum has a CPK
color and a radius for use as convenient defaults when representing a
Bond as a cyliner.

---

**Enum Constant Summary**

`DISULFIDE`
  
          CPKColorEnum.SULPHUR\_YELLOW, alpha = 1.0, and radius = 0.2.

`DOUBLE`
  
          CPKColorEnum.LIGHT\_GRAY, alpha = 1.0, and radius = 0.2.

`HYDROGEN`
  
          CPKColorEnum.RED, alpha = 1.0, and radius = 0.2.

`PEPTIDE`
  
          CPKColorEnum.LIGHT\_GRAY, alpha = 1.0, and radius = 0.2.

`SINGLE`
  
          CPKColorEnum.LIGHT\_GRAY, alpha = 1.0, and radius = 0.2.

`TRIPLE`
  
          CPKColorEnum.LIGHT\_GRAY, alpha = 1.0, and radius = 0.2.


| **Method Summary** | |
| --- | --- |
| `float` | `getAlpha()`             Returns the alpha component of the RGBA color on a scale from 0.0 to 1.0, inclusive. |
| `float` | `getBlue()`             Returns the blue component of the RGBA color on a scale from 0.0 to 1.0, inclusive. |
| `float` | `getGreen()`             Returns the green component of the RGBA color on a scale from 0.0 to 1.0, inclusive. |
| `double` | `getRadius()`             Returns a radius that can be used for representing the Bond as a cylinder. |
| `float` | `getRed()`             Returns the red component of the RGBA color on a scale from 0.0 to 1.0, inclusive. |
| `static BondEnum` | `valueOf(String name)`             Returns the enum constant of this type with the specified name. |
| `static BondEnum[]` | `values()`             Returns an array containing the constants of this enum type, in the order they're declared. |

**Methods inherited from class java.lang.Enum**

`clone, compareTo, equals, getDeclaringClass, hashCode, name, ordinal, toString, valueOf`

**Methods inherited from class java.lang.Object**

`finalize, getClass, notify, notifyAll, wait, wait, wait`

**Enum Constant Detail**

### SINGLE

```
public static final BondEnum SINGLE
```

:   CPKColorEnum.LIGHT\_GRAY, alpha = 1.0, and radius = 0.2.
    Single covalent bond between two atoms.

---


### DOUBLE

```
public static final BondEnum DOUBLE
```

:   CPKColorEnum.LIGHT\_GRAY, alpha = 1.0, and radius = 0.2.
    Double covalent bond between two atoms.

---


### TRIPLE

```
public static final BondEnum TRIPLE
```

:   CPKColorEnum.LIGHT\_GRAY, alpha = 1.0, and radius = 0.2.
    Triple covalent bond between two atoms.

---


### PEPTIDE

```
public static final BondEnum PEPTIDE
```

:   CPKColorEnum.LIGHT\_GRAY, alpha = 1.0, and radius = 0.2.
    The bond formed when the carboxyl group of one amino
    acid reacts with the amino group of another amino acid.

---


### DISULFIDE

```
public static final BondEnum DISULFIDE
```

:   CPKColorEnum.SULPHUR\_YELLOW, alpha = 1.0, and radius = 0.2.
    A strong covalent bond between two sulfhydryl groups
    (for example, the bond between two cysteine residues
    in a protein).

---


### HYDROGEN

```
public static final BondEnum HYDROGEN
```

:   CPKColorEnum.RED, alpha = 1.0, and radius = 0.2.
    A weak bond between two partial electric charges, where
    the partial positive charge is on a hydrogen atom.


**Method Detail**

### values

```
public static final BondEnum[] values()
```

:   Returns an array containing the constants of this enum type, in
    the order they're declared. This method may be used to iterate
    over the constants as follows:

    ```
    for(BondEnum c : BondEnum.values())
            System.out.println(c);
    ```

    :   **Returns:**: an array containing the constants of this enum type, in the order they're declared

---


### valueOf

```
public static BondEnum valueOf(String name)
```

:   Returns the enum constant of this type with the specified name.
    The string must match *exactly* an identifier used to declare an
    enum constant in this type. (Extraneous whitespace characters are
    not permitted.)

    :   **Parameters:**: `name` - the name of the enum constant to be returned. **Returns:**: the enum constant with the specified name **Throws:**: `IllegalArgumentException` - if this enum type has no constant with the specified name

---


### getRed

```
public float getRed()
```

:   Returns the red component of the RGBA color on a scale from 0.0
    to 1.0, inclusive.

    :   **Returns:**: The red value as a float.

---


### getGreen

```
public float getGreen()
```

:   Returns the green component of the RGBA color on a scale from 0.0
    to 1.0, inclusive.

    :   **Returns:**: The green value as a float.

---


### getBlue

```
public float getBlue()
```

:   Returns the blue component of the RGBA color on a scale from 0.0
    to 1.0, inclusive.

    :   **Returns:**: The blue value as a float.

---


### getAlpha

```
public float getAlpha()
```

:   Returns the alpha component of the RGBA color on a scale from 0.0
    to 1.0, inclusive.

    :   **Returns:**: The alpha value as a float.

---


### getRadius

```
public double getRadius()
```

:   Returns a radius that can be used for representing the Bond as a
    cylinder.

    :   **Returns:**: The radius value as a double.


---


|  |  |  |
| --- | --- | --- |
| **Overview** \| **Package** \| **Class** \| **Use** \| **Tree** \| **Deprecated** \| **Index** \| **Help** | |  |
| **PREV CLASS**   **NEXT CLASS** | **FRAMES**    **NO FRAMES**     **All Classes** |  |
| SUMMARY: NESTED \| ENUM CONSTANTS \| FIELD \| METHOD | DETAIL: ENUM CONSTANTS \| FIELD \| METHOD |  |


---

# *Copyright © 2007-2008*
